# Age-related impairment of humoral response to influenza is associated with changes in antigen specific T follicular helper cell responses

**DOI:** 10.1038/srep25051

**Published:** 2016-04-25

**Authors:** Julie S Lefebvre, April R Masters, Jacob W Hopkins, Laura Haynes

**Affiliations:** 1 Trudeau Institute, 154 Algonquin Ave, Saranac Lake, NY, 12983, USA; 2 University of Connecticut School of Medicine, 263 Farmington Ave, Farmington, CT 06030, USA.

## Abstract

T follicular helper (T_FH_) cell responses are essential for generation of protective humoral immunity during influenza infection. Aging has a profound impact on CD4^+^ T cell function and humoral immunity, yet the impact of aging on antigen specific T_FH_ responses remains unclear. Influenza specific T_FH_ cells are generated in similar numbers in young and aged animals during infection, but T_FH_ cells from aged mice exhibit significant differences, including reduced expression of ICOS and elevated production of IL-10 and IFNγ, which potentially impairs interaction with cognate B cells. Also, more influenza specific T cells in aged mice have a regulatory phenotype, which could contribute to the impaired T_FH_ function. Adoptive transfer studies with young T cells demonstrated that TGF-β1 in the aged environment can drive increased regulatory T cell accumulation. Aging and the aged environment thus impact antigen specific T_FH_ cell function and formation, which contribute to reduced protective humoral responses.

Aging is associated with a dramatic decline in immunity including production of high affinity neutralizing antibodies^1^, which leads to increased susceptibility to clinically relevant pathogens such as influenza. High affinity antibodies are generated by B cells selected in germinal centers (GC) and GC formation is dependent upon the proper function of T follicular helper (T_FH_) cells^2^,^3^,^4^,^5^. Following initial activation, naïve CD4^+^ T cells progress through several well defined steps to become functional T_FH_ cells in GCs^6^,^7^. First, pre-T_FH_ cells up-regulate the B cell follicle homing chemokine receptor CXCR5 and the transcriptional repressor B cell lymphoma 6 (Bcl6). These cells also produce IL-4 and IL-21, key cytokines of B cell activation. This first stage is the result of T-dendritic cell (DC) interactions and is independent of B cells^8^. The activated T cells then progress toward a fully differentiated T_FH_ cell state characterized by the expression of numerous markers including the inhibitory receptor programmed death (PD)-1, the co-stimulatory molecules inducible T-cell costimulator (ICOS) and OX40, along with the adaptor protein signaling lymphocytic activation molecule (SLAM)-associated protein (SAP)^9^. These molecules facilitate efficient T-B interactions, which are essential for GC formation. Deficiencies in ICOS^8^,^10^ or SAP^11^,^12^ result in reduced T_FH_ cell generation and/or reduced GC formation. These interactions with B cells induce the further differentiation of T_FH_ cells into GC T_FH_ cells, which are identifiable through the sustained expression of GL7 Akiba *et al.*^10^,^13^,^14^,^15^. Importantly, how aging impacts the stages of T_FH_ cell generation and differentiation is yet to be determined.

The presence of functional defects in aged CD4^+^ T cells is now well-established^16^. We demonstrated that aged CD4^+^ T cells exhibit impaired upregulation of CD154^17^, suggesting that reduced B cell activation and humoral responses observed in aged mice could result from improper T-B interactions. Differentiation of young CD4^+^ T cells into T_FH_ effectors cells was also significantly reduced when these cells were transferred into aged hosts^18^, suggesting that the aged environment is unable to sustain T_FH_ cell differentiation. These observations were made in adoptive transfer models using T cell receptor (TCR) transgenic CD4^+^ T cells. Whether endogenous antigen-specific aged CD4^+^ T cells have the ability to acquire a T_FH_ cell phenotype and/or whether endogenous aged T_FH_ cells have functional defects during infection remains unclear. A recent study using an OVA immunization model showed that aged mice have more T_FH_ than young mice but aged T_FH_ were less functional using *in vitro* assays^19^. This study did not track antigen specific responses *in vivo*, and did not thoroughly define the T_FH_ maturation status. Moreover, the capacity of aged mice to mount an antigen-specific CD4^+^ T cell response equivalent to that of young mice and whether it correlates with an antigen-specific B cell response remains to be investigated. Indeed, these are important points and only when they are fully addressed can we begin to understand the age-related defects in humoral immunity and, more importantly, how they can be overcome.

Here, we have employed reagents that allow us to follow the responses of endogenous influenza nucleoprotein (NP)-specific CD4^+^ T and B cells in young and aged mice. Studying endogenous antigen specific responses to infection is critical to understanding the full scope of immunological defects that occur with age. This has allowed us to determine specific defects in responding T_FH_ cells in aged mice and their impact on humoral responses.

## Results

### Impaired humoral response in aged mice following influenza infection

**Figure 1:**
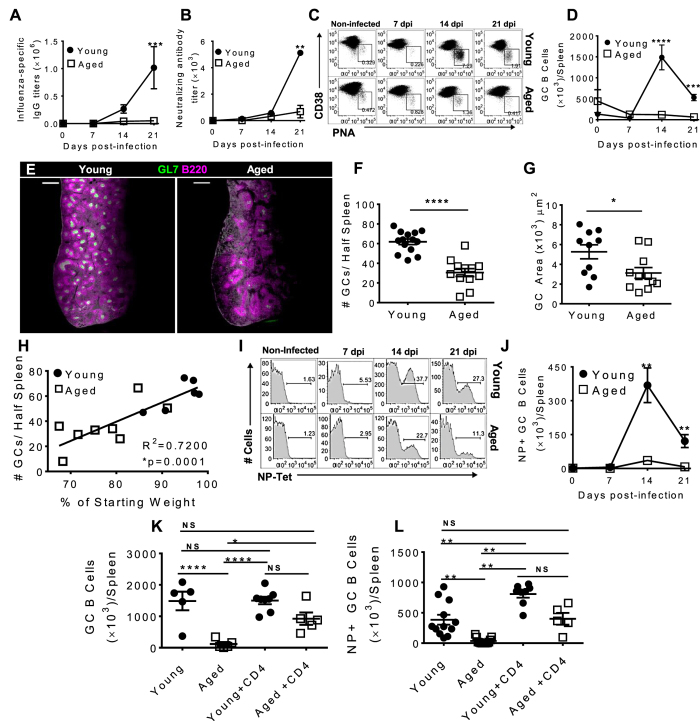
Reduced antibody production and GC development during influenza infection in aged mice. (**A**) Influenza-specific IgG titers, and (**B**) influenza-neutralizing antibody titers in young and aged mice infected with 600 PFU of PR8 at 0, 7, 14 and 21 dpi. Data are the mean ± SEM of 4–5 mice/group from a representative experiment of 4 independent experiments. (**C**) Kinetics of CD38/PNA expression following influenza infection after gating on live CD19^+^ lymphocytes. The gates delineate the GC B cell population (PNA^hi^ CD38^lo^) with numbers adjacent representing frequency of GC B cells. The data are 5 mice/group from a representative experiment of 3 performed independently. (**D**) Total number of splenic GC B cells determined using the gating strategy shown in (**C**). Data are the mean ± SEM of 8–13 mice/group from three independent experiments. (**E**) Representative half spleen images from young and aged mice 14 dpi, GL7 (green) B220 (magenta) with scale bar at 500μm. GC numbers per half spleen (**F**) and GC area (**G**) quantified by confocal microscopy. (**H**) Correlation of splenic GC number and weight recovery at 14 dpi. Data are the mean ± SEM from 3 combined experiments with 3–4 mice per group. (**I**) Frequency of NP-specific GC B cells (gated as in (**C**)). Histograms show data of 4–5 mice/group from one representative experiment of two performed independently. (**J**) Total splenic NP-specific GC B cells determined using the combined gating strategies shown in (**C**,**I**). Data are the mean ± SEM of 8–13 mice/group from three independent experiments. Number of total (**K**) or NP-specific (**L**) GC B cells at 14 dpi in young or aged mice that received/did not receive 3.6 × 10^7^ young CD45.1^+^CD4^+^ T cells one day prior to infection with PR8, gated on CD45.1- GC B cells as in (**C,I**). Data are the mean ± SEM of 4–7 mice per group from a representative experiment of 2 performed independently. Statistical significance was determined by (**A,B,D,J**) two-way ANOVA (Bonferronni’s post hoc), (**F**,**G**), Student’s t test, (**H**) Pearson’s correlation and (**K,L**) one-way ANOVA (Tukey HSD post hoc). *p < 0.05,**p < 0.01; ***p < 0.001; ****p < 0.0001.

One hallmark of aging is the inability to mount a strong humoral response. Aged mice infected with a sublethal dose (600 PFU) of influenza A H1N1 PR8 generated significantly lower titers of influenza-specific IgG (Fig. 1A) and neutralizing antibodies (Fig. 1B) compared to young. Generation of high-affinity antibodies is dependent upon formation of GCs, which can be impacted by age-related functional declines^20^. Thus, we sought to investigate the contribution of GCs to diminished formation of influenza-specific antibody titers in aged mice in this model.

GC B cells were identified as CD19^+^ PNA^hi^ CD38^lo^ cells (boxes in Fig. 1C). The kinetics of total (Fig. 1C,D) and NP-specific (Fig. 1I,J) GC B cell generation were similar for both young and aged mice, with the proportion and number peaking at 14 days post-infection (dpi). However, the amplitude of the response was significantly lower in aged compared to young mice. At 14 dpi, approximately 1% of total B cells acquired a GC B cell phenotype in aged mice compared to over 7% in young mice (Fig. 1C). This translated to a significantly lower number of total GC B cells produced in the spleens of aged compared to young mice (Fig. 1D).

Upon examination of the splenic GCs using confocal microscopy, there were clear morphological differences in young and aged mice (Fig. 1E). At the peak of the GC response (14 dpi), aged mice exhibited significantly fewer (Fig. 1F) and smaller GCs (Fig. 1G) compared to young mice. Importantly, there was a strong correlation between the number of splenic GCs and weight recovery at 14 dpi (Fig. 1H). Young and aged mice that generated more splenic germinal centers had increased weight recovery after influenza infection (Fig. 1H), indicating that rapid recovery from influenza infection is correlated with robust GC formation. The percentage of GC B cells that were specific for NP was slightly, but not significantly, lower in aged (22.7 ± 7.2%) compared to young mice (37.7 ± 3.9%, p = 0.104) (Fig. 1I) at 14 dpi, but the total number of NP^+^ GC B cells in aged mice was approximately ten fold lower than in young mice (Fig. 1J).

Despite age related declines in B cell function^21^, young polyclonal CD4^+^ T cells transferred into aged hosts can overcome the defects in GC B cell numbers normally found in aged mice at 14dpi. When aged mice were supplemented with young CD4^+^ T cells prior to infection (aged + CD4 group), the number of total (Fig. 1K) and NP specific (Fig. 1L) GC B cells reaches that of the endogenous response of young mice (young group) at 14 dpi, emphasizing the importance of efficient CD4^+^ T cell help in the development of a robust B cell response. These results are in concordance with our previous finding that young TCR transgenic CD4^+^ T cells can overcome B cell defects in aged CD4 KO mice^17^. Thus, the remainder of this study was aimed at determining how age-related defects in the CD4^+^ T cell response contribute to the impaired humoral response in aged mice.

### Aged influenza-specific CD4^+^ T cells differentiate into pre-T_FH_ cells

**Figure 2:**
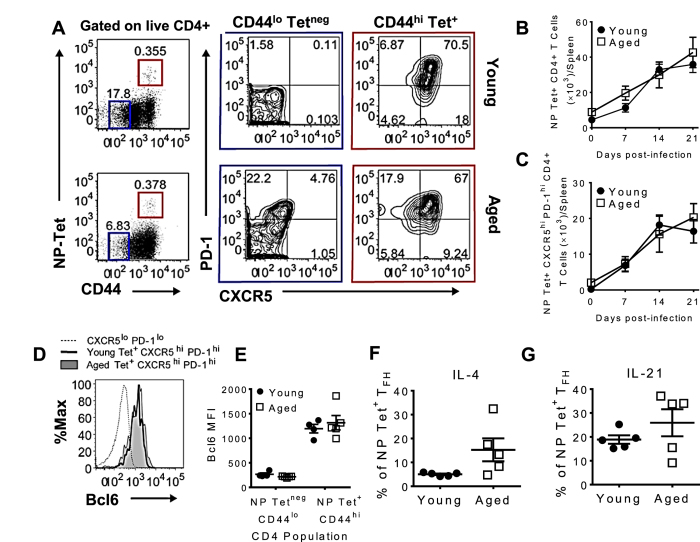
Aged CD4^+^ T cells differentiate into pre-T_FH_ cells. (**A**) CXCR5/PD-1 expression of young (top row) and aged (bottom row) Tet^+^ CD44^hi^ (red boxes) and Tet^neg^ CD44^lo^ (blue boxes) cells after gating on live CD4^+^ T lymphocytes at 14 dpi. Plots show concatenated data of 5 mice/group from one representative experiment of three performed independently. (**B**) Total number of splenic NP-specific CD4^+^ T cells determined by flow cytometry using the gating strategy shown in (**A**). Data are the mean ± SEM of 8–13 mice/group pooled from three independent experiments. (**C**) Total number of splenic Tet^+^ CXCR5^hi^ PD-1^hi^ in young and aged mice determined by flow cytometry using the gating strategy shown in (**A**). Data are the mean ± SEM of 8–13 mice/group pooled from three independent experiments. (**D**) Bcl6 expression by young and aged NP-specific CXCR5^hi^ PD-1^hi^ CD4^+^ T cells at 14 dpi. Negative expression (dashed line) was determined using young CXCR5^lo^ PD-1^lo^ CD4 T cells. The histogram represents concatenated data of 5 mice/group from one representative experiment of three performed independently. (**E**) Bcl6 MFI shown in (**D**). Frequency of young and aged NP Tet^+^ CD4^+^ T cells producing (**F**) IL-4, and (**G**) IL-21 following PMA and ionomycin stimulation at 7 dpi. The data show the mean ± SEM of 5 mice/group from one representative experiment of two performed independently. Statistical significance was determined by Student’s t test.

To determine how the antigen-specific CD4^+^ T cell response is affected by aging, we used a MHC Class II influenza nucleoprotein (NP)-specific tetramer to track cells in young and aged mice following infection. Figure 2A shows a representative gating strategy for phenotyping young (top row) and aged (bottom row) NP-specific CD4^+^ T cells 14 dpi. The kinetics and number of NP-specific CD4^+^ T cells accumulating in the spleen of young and aged mice following influenza infection were equivalent (Fig. 2B). Analysis of NP-specific CD4^+^ T cells showed that most (~70%) displayed a T_FH_ cell phenotype (CXCR5^hi^ PD-1^hi^) at 14 dpi (Fig. 2A, red boxes), but also at 7 and 21 dpi (data not shown). The numbers of NP-specific T_FH_ cells were also equivalent in both young and aged mice at all time-points tested (Fig. 2C). Also of note, a higher percentage of NP-Tet^neg^ CD44^lo^ CD4^+^ T cells in aged mice express PD-1 compared to young mice (Fig. 2A blue box), a feature characteristic of aging cells^22^,^23^. Total T_FH_ responses did not follow the trend of the antigen-specific response, with aged mice having a higher frequency and number of T_FH_ than young mice before infection, 7 dpi and 21 dpi (Supplemental Fig. 1). Young mice responded to influenza infection with an expansion of T_FH_ that peaked at day 14 post infection (Supplemental Fig. 1). Aged mice had relatively stable T_FH_ numbers and frequency through the infection (Supplemental Fig. 1), demonstrating that total response is not representative of the antigen-specific response occurring in aged mice.

To confirm the identity of the CXCR5^hi^ PD-1^hi^ NP-specific CD4^+^ T cells as T_FH_ cells, we assessed their expression of Bcl6, the central driver of T_FH_ cell differentiation^24^. Bcl6 expression in both young and aged NP-specific T_FH_ cells was compared to the CXCR5^lo^ PD-1^lo^ CD4^+^ population (Fig. 2D). The Bcl6 mean fluorescence intensities (MFI) of young and aged NP-specific T_FH_ cells were not significantly different (Fig. 2E). To further confirm that both young and aged NP-specific CD4^+^ T cells were differentiating into T_FH_ cells, we assessed their ability to produce IL-4 and IL-21, cytokines produced by T_FH_ cells that are important for GC generation. Due to the limitation in the number of markers that could be used at once for flow cytometry analyses, and since the great majority of the NP-specific CD4^+^ T cells displayed T_FH_ characteristics (Fig. 1A), we considered that the splenic NP-specific CD4^+^ T cells were “T_FH_-committed” cells for the remainder of these studies. In agreement with the differentiation of CD4^+^ T cells to a T_FH_ cell phenotype, both young and aged NP-specific CD4^+^ T cells produced IL-4 (Fig. 2F) and IL-21 (Fig. 2G) in similar proportions. Thus, our data demonstrate that age does not impair the ability of CD4^+^ T cells to differentiate into pre-T_FH_ cells (CXCR5^+^ Bcl6^+^ CD4^+^ T cells)^6^. Since our previous data using aged TCR transgenic cells showed that aged CD4^+^ T cells exhibit defective helper function^17^, we hypothesized that defects in the later stages of T_FH_ cell differentiation may be impaired in aged NP-specific CD4^+^ T cells.

### Aged influenza-specific CD4^+^ T cells have impaired differentiation to a mature T_FH_ cell phenotype

**Figure 3:**
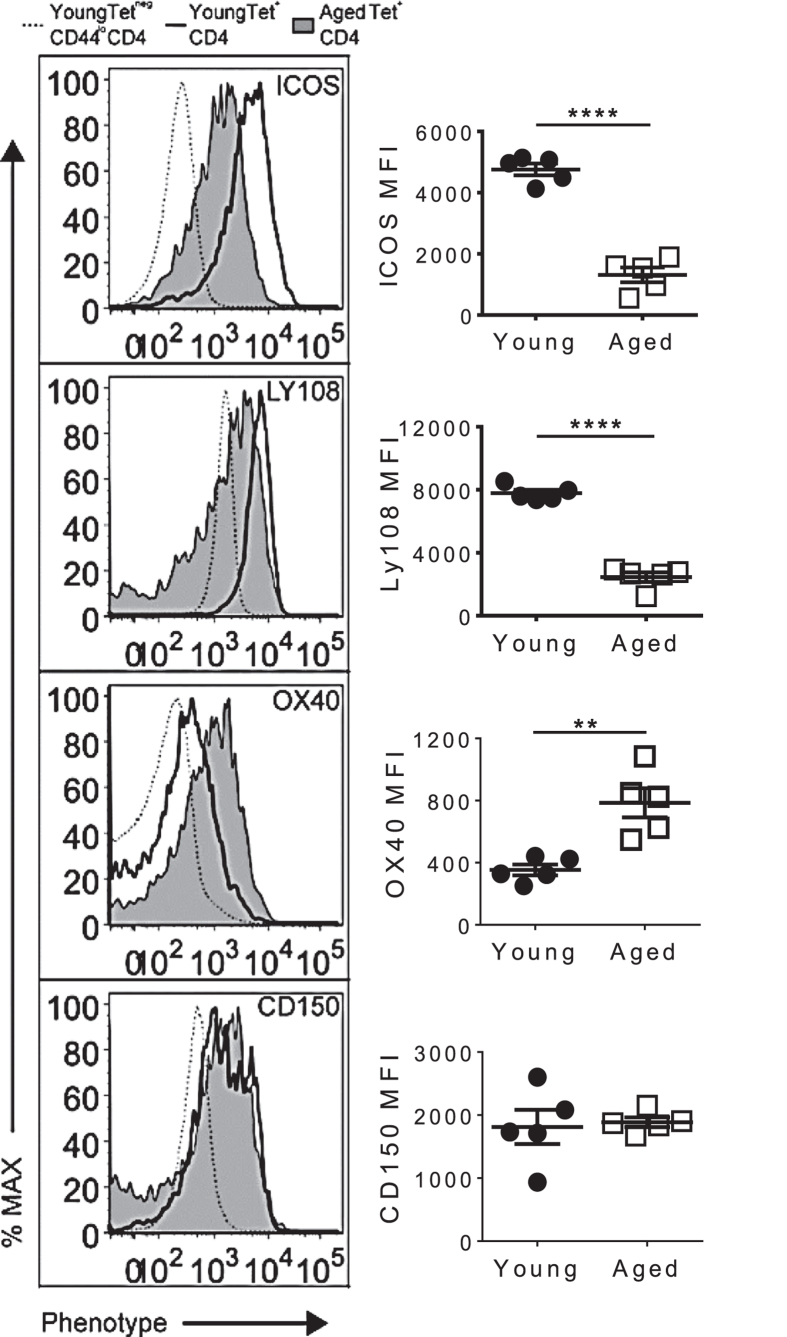
Aged CD4^+^ T cells fail to progress to fully differentiated T_FH_ cells. (Left) Histograms showing ICOS, Ly108, OX40 and CD150 expression by young and aged NP Tet^+^ CD4^+^ T cells at 7 dpi. Negative or baseline expression (dashed line) was determined using the young Tet^neg^ CD44^lo^ CD4 T cell population. (Right) MFI of the histograms shown on left. The histograms (Left) show concatenated data, and the graphs (Right) show the mean ± SEM, of 5 mice/group from one representative experiment of two performed independently. Statistical significance was determined by Student’s t test. **p < 0.01 ****p < 0.0001.

Following the pre-T_FH_ stage, cells upregulate the expression of surface markers involved in T-B cell interactions, and migrate to the T-B cell border where their initial interaction with B cells occurs. To determine if aged NP-specific T cells express the appropriate cell surface molecules to interact efficiently with B cells, we evaluated expression of ICOS, Ly108, OX40 and CD150 on young and aged NP-specific T cells (Fig. 3). ICOS and Ly108 surface expression on aged NP-specific CD4^+^ T cells were significantly lower than on young CD4^+^ T cells. Conversely, the expression of OX40 on aged NP-specific CD4^+^ T cells was significantly higher. CD150 expression was similar on both young and aged cells.

These data suggests that aged NP-specific CD4^+^ T cells may have impaired ability to interact with B cells due to their failed ability to appropriately up-regulate ICOS and Ly108. Since ICOS and Ly108 play an important role in T-B interactions and GC formation^10^,^25^, lower expression of these molecules may contribute to the functional defects of aged CD4^+^ T cells which in turn may contribute to the reduced humoral responses observed in aged mice.

**Figure 4:**
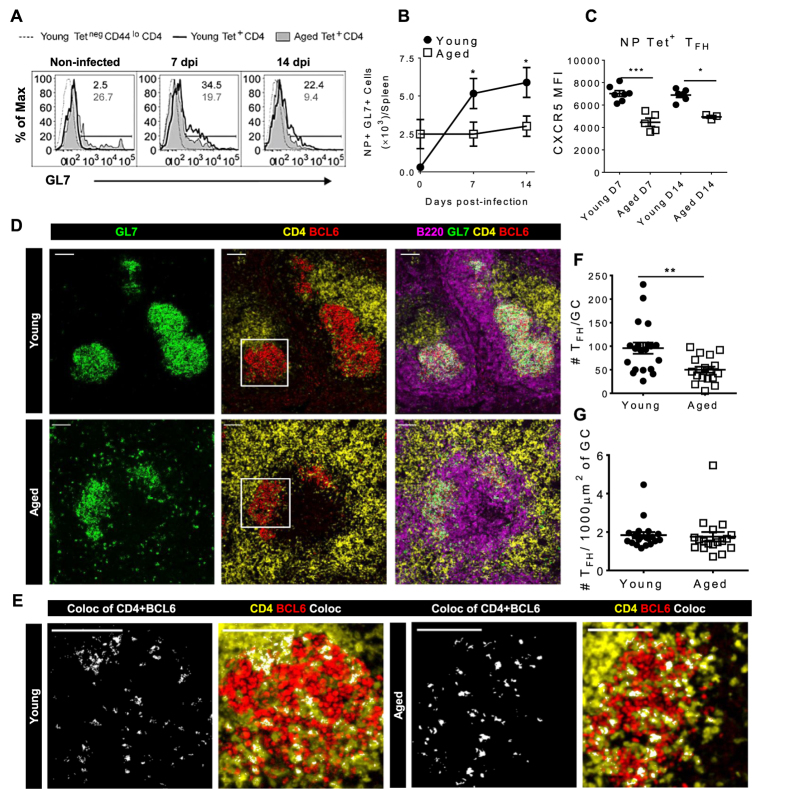
Decreased germinal center T_FH_ cell response to influenza infection in aged mice (**A**) GL7 expression on young and aged NP Tet^+^ CD4^+^ T cells. Negative expression (dashed line) was determined using young NP Tet^neg^ CD44^lo^ CD4^+^ cells. The histograms show concatenated data of 4–5 mice/group from one representative experiment of 3 performed independently. Numbers in the upper right corners are the frequency of the young (black) and aged (grey) cells in the GL7^+^ gate. (**B**) Total number of splenic NP Tet^+^ GL7^+^ cells determined by flow cytometry using the gating strategy shown in (**A**). Data represents the mean ± SEM of 14**–**18 mice/group from 3 independent experiments. (**C**) CXCR5 MFI of NP Tet^+^ T_FH_ cells from young and aged mice at 7 and 14 dpi (using gating strategy from Fig. 2A). Data represents the mean ± SEM of 3–7 mice/group from 3 independent experiments. (**D**) Representative images of spleens from young (top row) and aged (bottom row) mice 14 dpi stained with GL7 (green) to denote GCs, CD4 (yellow), Bcl6 (red) and B220 (magenta) with scale bars at 80μm. Inserts in the middle panel are magnified in figure (**E**). (**E**) T_FH_ cells were characterized by co-localization of Bcl6 and CD4 (white). (**F**) T_FH_ cells per GC were enumerated by counting the T_FH_ cells in the GL7 area using Imaris software. (**G**) T_FH_ cells per 1000 μm^2^ GC area were calculated using the GC area (determined by creating an isosurface of GL7 with Imaris) and the number of T_FH_ cells per GC. Data are mean ± SEM pooled from three independent experiments with images from 17 mice containing 1–4 GC per image (**D–F**). Statistical significance was determined by Student’s t test. *p < 0.05; **p < 0.01, ***p < 0.001.

The last stage of T_FH_ cell differentiation corresponds to entry into the GC. This is characterized by the expression of GL7, a marker expressed by T_FH_-committed CD4^+^ T cells^13^ and associated with the mature GC T_FH_ cell phenotype^14^. Considering that the NP-specific pre-T_FH_ have altered receptor expression that may impair their interaction with cognate B cells, we expected that the progression of the aged NP-specific CD4^+^ T cells to the final GC T_FH_ cell phenotype may also be impaired. We therefore evaluated whether aged T_FH_ cells were progressing normally to the GC T_FH_ cell phenotype by evaluating GL7 expression on these cells. While 34.5% of the young NP-specific CD4^+^ T cells up-regulated GL7 at 7 dpi, only 19.7% of the aged cells were GL7 positive at that time-point (Fig. 4A). This resulted in a significantly higher number of GC NP-specific CD4^+^ T_FH_ cells in young compared to aged mice which were maintained at 14 dpi (Fig. 4B). To further explore the incomplete differentiation of aged antigen-specific T_FH_ cells to a GC T_FH_ cell phenotype, we analyzed the expression of CXCR5, an important molecule for the migration of pre-T_FH_ cells into the GC^3^. The aged NP-specific T_FH_ cells exhibited lower expression of CXCR5 compared to their young counter parts (Fig. 4C), corresponding to a pre-T_FH_ cell phenotype^14^.

Our data thus far indicate that aged influenza-specific CD4^+^ T cells differentiate normally to a pre-T_FH_ cell phenotype, but less progress to a mature GC phenotype compared to young mice. To visualize this immunological reaction *in situ* we used multi-color confocal microscopy. Due to the limitations of imaging with MHC class II tetramers, we focused on visualizing the total response to influenza infection in young and aged spleens. There was increased GC disorganization, noted by the scattered GL7^+^ areas (Fig. 4D, left panels). The merged images demonstrate that aged mice had notable disruption of their splenic white pulp architecture when compared to young, denoted by the merging of the T and B cell areas (Fig. 4D, right panels). The considerable disruption of the microarchitecture observed in GCs from aged mice could also contribute to the reduced production of a protective humoral response. Next we sought to determine if T_FH_ localize to the germinal center comparably in aged and young mice. T_FH_ cells were identified by co-localization of Bcl6 and CD4 (Fig. 4E, insert from Fig. 4D middle panel). To quantify GC T_FH_ cells, the cells expressing CD4 and Bcl6 in the GL7^+^ areas were counted. Aged mice had fewer T_FH_ cells per GC compared to young mice at 14 dpi (Fig. 4F) in concordance with our antigen-specific T_FH_ flow cytometry data (Fig. 4B). However, since the size of each GC is smaller in area in aged mice when compared to GC in young mice (Fig. 1G), the number of T_FH_ per 1000 μm^2^ of GC is not different between young and aged mice (Fig. 4G). From this data we can conclude that the decreased number of T_FH_ in the germinal center is a function of the decreased germinal center size, not density.

Taken together, these data support the hypothesis that the activation and differentiation of the aged NP-specific CD4^+^ T cells in response to influenza infection is impaired and results in lower numbers of GC T_FH_ cells that may contribute to a deficient GC response. We then interrogated the expression of PD-1 on young and aged CD4^+^ T cells during influenza infection since increased PD-1 expression on aged T_FH_ was described recently by Sage *et al.*^19^, who suggested that increased PD-1 expression could be responsible for the reduced function of aged T_FH._ The total aged CD4^+^ T cell population (Supplemental Fig. 2A–C) as well as the total aged T_FH_ population (Supplemental Fig. 2D,E) do indeed express higher levels of PD-1 when compared to young. Importantly, and in contrast to the report by Sage *et al.*, there is no difference in PD-1 expression on NP-specific T_FH_ from young and aged mice at 7 dpi (Supplemental Fig. 2F) and by 14 dpi, young T_FH_ express higher levels when compared to aged (Supplemental Fig. 2G). Thus, in our influenza model, increased expression of PD-1 by aged Ag-specific T_FH_ cannot account for their reduced function.

### NP-specific CD4^+^ T cells from aged mice have a regulatory-like phenotype

**Figure 5:**
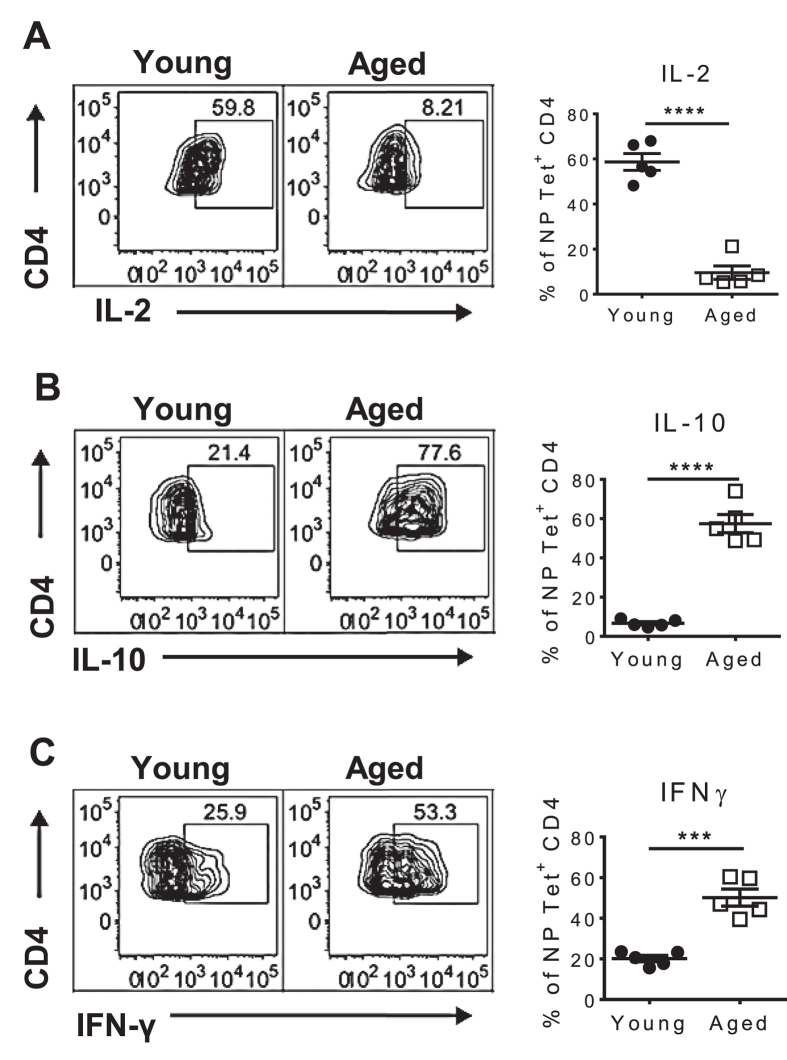
NP-specific CD4^+^ T cells produce cytokines indicative of regulatory T cells. Intracellular cytokine staining of young and aged NP Tet^+^ CD4^+^ T cells harvested 7 dpi and stimulated with PMA/Ionomycin. The graphs show the mean ± SEM of the frequency of NP-specific CD4^+^ T cells producing: (**A**) IL-2, (**B**) IL-10, and (**C**) IFNγ. The data are from 5 mice/group of one representative experiment of two performed independently. Statistical significance was determined by Student’s t test. ***p < 0.001; ****p < 0.0001.

Since the progression of CD4^+^ T cells into fully differentiated GC T_FH_ cells is impaired in aged mice, we hypothesized that their B cell helper activity would be affected. In the next series of experiments we evaluated the IL-2, IL-10 and IFNγ production by young and aged NP-specific CD4^+^ T cells by flow cytometry. Consistent with previous reports^26^, the proportion of aged NP-specific CD4^+^ T cells producing IL-2 was significantly lower than that of young cells (Fig. 5A). Interestingly, a higher frequency of aged cells produced IL-10 (Fig. 5B) and IFNγ (Fig. 5C) than young cells. The dysregulated cytokine production by aged NP-specific CD4^+^ T cells could indicate an inappropriate effector differentiation program that might impact helper activity of these cells, and therefore, the formation of GCs.

**Figure 6:**
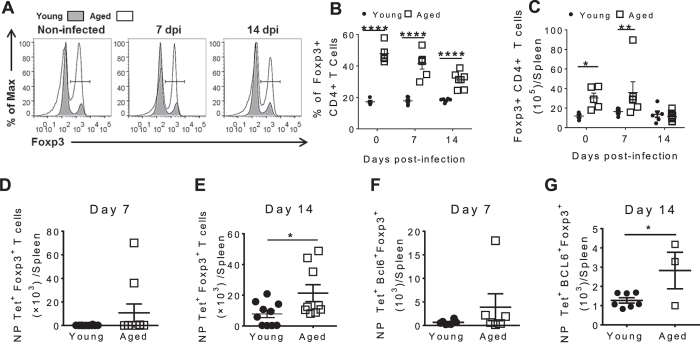
T regulatory and T follicular regulatory cells accumulate in aged mice after influenza infection. (**A**) Representative histograms of total CD4^+^ T cells expressing Foxp3 in young and aged mice. Frequency (**B**) and number (**C**) of total CD4^+^ T cells that express Foxp3 in spleens from young and aged mice before influenza infection, 7dpi and 14dpi. Number of NP-specific CD4^+^ T cells expressing Foxp3 in the spleen of young and aged mice at (**D**) 7 dpi and (**E**) 14 dpi. Number of NP-specific CD4^+^ CXCR5^hi^ PD-1^hi^ Bcl6^+^ Foxp3^+^ cells in spleens of young and aged mice at (**F**) 7 dpi and (**G**) 14 dpi. Data are the mean ± SEM of 3–10 mice per group from a representative experiment of 3 performed independently. Statistical significance was determined by Two way ANOVA with LSD post test (**B,C**) or Student’s t test (**D–G**). *p < 0.05, p < 0.001.

Reduced IL-2 production combined with increased IL-10 production is reminiscent of a regulatory T cell phenotype and it is reported that the frequency of regulatory T cells increases with aging^27^,^28^,^29^,^30^. Upon examination of total Foxp3^+^CD4^+^ T Cells our data also showed increased frequency of regulatory T cells in aged mice at all time points test (Fig. 6A,B). Total number of regulatory T cells was only significantly higher in aged mice at steady state and 7 dpi (Fig. 6C). To determine if more aged NP-specific CD4^+^ T cells exhibited regulatory T cell properties, we evaluated Foxp3 expression in young and aged influenza infected mice. At 7 dpi, the number of NP-specific CD4^+^ T cells expressing Foxp3 was not significantly different in young and aged groups (Fig. 6A). By 14 dpi however, the number of NP-specific CD4^+^ T cells expressing Foxp3 was significantly higher in aged compared to young mice (Fig. 6B). Recently, a subset of regulatory T cells that specifically control GC B cell and GC T_FH_ cell responses, T follicular regulatory (T_FR_) cells, was characterized. T_FR_ cells are identified by expression of CD4, PD-1, CXCR5, Foxp3 and Bcl6^31^,^32^,^33^. At 7 dpi there was no difference in NP-specific T_FR_ cell formation (Fig. 6F), but at the peak of the GC response (14 dpi) aged mice had increased numbers of NP-specific T_FR_ cells (Fig. 6G). Total T_FR_ cell number was also increased aged mice compared to young mice at all time points post infection (Supplemental Fig. 1). The increase in NP-specific T_FR_ cells and regulatory T cells may contribute to the poor GC T_FH_ cell and humoral response observed in aged mice after influenza infection. In order to further understand how aging impacts regulatory cells, we next explored the contribution of the aged environment to their formation.

### Impact of the aged environment on regulatory T cells

**Figure 7:**
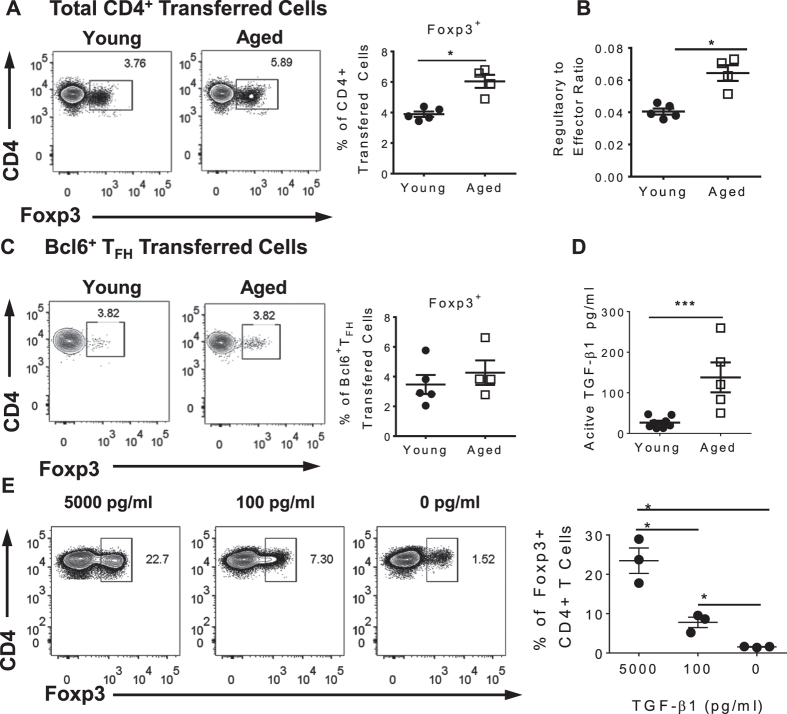
More young CD4^+^ T cells transferred into aged host express Foxp3 compared to young hosts. Purified young CD45.1^+^CD4^+^ polyclonal T cells were transferred via tail vein injection into young or aged hosts at 3.6 × 10^7^ cells per mouse. One day post transfer, recipient mice were infected with 600PFU PR8 influenza and subsequently sacrificed for analysis 14 days later. (**A**) Foxp3 expression of CD4^+^ transferred cells in young or aged hosts. (**B**) Regulatory to effector ratio of CD4^+^ transferred cells in young or aged host, calculated by the percent of Foxp3^+^/Foxp3^−^ donor CD4^+^ cells. (**C**) CD4^+^, CXCR5^hi^, PD-1^hi^, Bcl6^+^ transferred cells expressing Foxp3 in young or aged host. (**D**) Active TGFβ-1 levels in spleen supernatants from the mice who received transferred cells described above. Data are the mean ± SEM of 4–7 mice per group from a representative experiment of 3 performed independently. Statistical significance was determined by Student’s t test (**A–D**). *p < 0.05. ***p < 0.001. (**E**) CD4^+^CD25^−^ T Cells from young mice were cultured with 5000 pg/ml (standard concentration for making iTregs^35^), 100 pg/ml (concentration in aged spleens) or 0 pg/ml TGF-β1. Combined results from three independent experiments. Statistical significance was determined using repeated measures ANOVA with Sidak post hoc test. *p < 0.05.

To assess whether the accumulation of regulatory T cells in aged mice was cell intrinsic or if the aged environment contributed to their accumulation, we performed an adoptive transfer experiment. Young and aged mice received young polyclonal CD45.1^+^ CD4^+^ T cells one day prior to influenza infection. We employed a polyclonal model for this experiment since T_FR_ cells are thymically derived and are not present in TCR transgenic populations such as the OT-II model^31^. The phenotype of the young CD4^+^ transferred T cells was analyzed by flow cytometry 14 dpi. A greater frequency of donor T cells in aged mice expressed Foxp3 when compared to donor cells in young mice (Fig. 7A). This was complemented by the increased regulatory to effector ratio of young CD4^+^ transferred cells in aged hosts (Fig. 7B). Examination of donor CD4^+^ T cells expressing a T_FR_ phenotype (Bcl6^+^ and Foxp3^+^) showed no difference in frequency in young or aged hosts (Fig. 7C). Although the factors that promote T_FR_ cell development are still being elucidated, TGF-β1 is a known inducer/stabilizer of Foxp3 expression^34^. There was a significantly higher level of active TGF-β1 in the spleens of aged mice compared to young mice at 14 dpi (Fig. 7D), which may contribute to accumulation of regulatory T cells in aged hosts. To determine whether the concentration of active TGF-β1 in the aged spleens was sufficient to promote regulatory T cell development, we performed an *in vitro* assay where young CD4^+^CD25^−^ T cells were cultured with either a standard concentration (5000 pg/ml) of TGF-β1 to polarize T cells to regulatory cells^35^, the concentration of TGF-β1 in aged spleens (100 pg/ml), or no TGF-β1. TGF-β1 at 100 pg/ml converted CD4^+^ T cells to regulatory T cells at a similar frequency (7%) to the *in vivo* transfer experiments (Fig. 7E), suggesting that TGF-β1 in the aged splenic environment can drive formation of regulatory T cells. These data suggest that there are both CD4^+^ T cell intrinsic and extrinsic factors that contribute to the regulatory environment of aged mice, which may negatively contribute to T_FH_ cell function and, in turn, dampen the humoral response in aged mice during influenza infection.

## Discussion

Aging is associated with a decline in immune system function, including reduced humoral responses. Here we show that low influenza-specific antibody titers found in aged mice following influenza infection are associated with decreased numbers of total and NP-specific GC B cells and decreased GC size, number, and responses compared to young mice. We have also shown that defects in total and NP-specific GC B cell numbers can be overcome by the addition of young CD4^+^ T cells. This suggests that B cell intrinsic defects are likely insufficient to explain the extent of the GC impairment and that other factors, such as age-related changed in T_FH_ cells, may contribute to these defects.

In previous studies, we have shown that aged CD4^+^ T cells acquire intrinsic defects that impair their helper functions^17^,^36^. These conclusions were drawn following the observation that aged TCR transgenic CD4^+^ T cells transferred into young CD4 KO mice support a weaker GC response when compared to young CD4^+^ T cells. This age-associated functional defect was correlated with impaired CD154 expression. Moreover, we have demonstrated that the aged microenvironment also contributes to reduced helper function of CD4^+^ T cells using an adoptive transfer model^18^. In these experiments, young CD4^+^ T cells displayed delayed activation and expansion, as well as reduced differentiation to a T_FH_ cell phenotype, when transferred into aged hosts when compared to young hosts^18^. This correlated with the impaired accumulation of the young donor cells in the T cell areas of the spleen in aged mice, a phenomenon likely related to the reduced expression of CCL19 and CCL21 in this organ^18^. These experiments allowed us to determine the contribution of the age-associated defects intrinsic and extrinsic to the CD4^+^ T cell compartment in the impaired GC response observed in aged mice.

Results presented herein provide evidence that endogenous, aged antigen-specific CD4^+^ T cells exhibit a defect in their ability to fully differentiate to a GC T_FH_ cell phenotype in response to influenza infection. While the same number of NP-specific CD4^+^ T cells acquired pre-T_FH_ characteristics (CXCR5^+^ PD-1^+^ Bcl6^+^) in young and aged mice, aged T_FH_ cells express lower levels of ICOS and Ly108 and fewer expressed GL7, a marker for GC T_FH_ cells. Importantly, this defect in T_FH_ cell differentiation is associated with impaired generation of NP-specific GC B cells. GL7 is a molecule readily expressed by activated NP-specific CD4^+^ T cells and maintenance of its expression in GC T_FH_ cells depends on the establishment of cognate T-B interactions^13^. Thus, we believe that the low frequency of aged NP-specific CD4^+^ T cells expressing GL7 is indicative of inadequate T-B cell interactions.

The hypothesis that aging negatively impacts T-B cell interactions is supported by several observations. First, the reduced number of NP-specific B cells in the spleen of aged mice likely contributes to the impaired GL7 expression. Second, this could be the result of impaired recruitment of T cells to B cell follicles. Our current findings are in agreement with our previous work showing that the architecture of B cell follicles in aged mice spleens was disorganized^18^. Moreover, the amount of CCR7 ligands CCL19 and CCL21 were significantly lower^18^, while the CXCR5 ligand CXCL13 was more abundant^37^ in aged compared to young mouse spleens. These signals may contribute to smaller GCs found in aged mice that have fewer T_FH_ cells than their young counterparts. Because of the imbalance of chemotactic signals and the disorganization of the splenic white pulp, the ability of aged T_FH_ cells to find and interact with their cognate B cells is likely compromised in aged mice.

Furthermore, we observed that expression of the co-stimulatory molecule ICOS was significantly reduced on aged compared to young NP-specific CD4^+^ T cells. A recent study by Xu and colleagues showed that ICOS participates in CD4^+^ T cell localization within B cell follicles in an ICOS ligand-independent manner^15^. A lower expression of ICOS on T_FH_ cells could reduce their ability to migrate within the follicles and interact with B cells. In concordance with these results, we also showed that NP-specific T_FH_ cells have lower expression of CXCR5, another important molecule for homing of T_FH_ cells to B cell follicles^3^. ICOS has a central role in multiple steps of the GC reaction, and ICOS deficient CD4^+^ T cells are unable to differentiate to a T_FH_ cell phenotype^10^. The equivalent generation of NP-specific T_FH_ cells in both young and aged mice, albeit with the lower expression of ICOS by the aged cells, suggests that this level of ICOS expression is sufficient for initial T_FH_ cell differentiation, but not for full differentiation to a GC T_FH_ cell phenotype. ICOS signaling also contributes to the GC response by inducing the expression of other co-stimulatory molecules such as CD40L^38^,^39^,^40^ and influencing cytokine production by T cells^38^,^39^,^40^ and GC B cells^41^. Ly108 has also been identified as a major regulator of T-B cell interactions^25^ through both positive and negative signals^42^. The reduced expression of both ICOS and Ly108 by the aged NP-specific CD4^+^ T cells could therefore have a major impact on the stability of the T-B cell interactions and the subsequent development of GCs.

Finally, we observed a greater number of NP-specific CD4^+^ T cells express the transcription factor Foxp3 in aged compared to young mice, with fewer aged cells producing IL-2 and more cells producing IL-10. Increased regulatory T cell frequency and/or number in aged mice have been reported by multiple groups^27^,^28^,^29^,^30^ and this increase has also been linked to reduced immune function^27^,^30^. Interestingly, the proportion of GL7^+^ GC T_FH_ cells is increased in mice deficient in regulatory T cells^43^. T_FR_ cells are important suppressors of GC B cell and T_FH_ cell responses that are derived from thymic regulatory T cells^31^,^32^,^33^. The increase in Foxp3^+^regulatory T cells found in aged mice could contribute to the increased number of total and antigen-specific T_FR_ cells found in aged mice. Sage *et al.* in a recent report demonstrated that young and aged T_FR_ cells have equal suppressive capacity. Therefore, T_FR_ cell from aged mice could contribute to reduced GC B cell and GC T_FH_ cell generation after infection. We have also demonstrated that the aged environment is conducive to regulatory T cell development, in part due to increased levels of active TGF-β1, a known inducer of Foxp3 expression^34^. Future studies will address the cellular source of TGF-β in aged mice.

In conclusion, we show that an appropriate number of NP-specific CD4^+^ T cells develop to pre-T_FH_ cell stage in aged mice. However, these cells are unable to fully progress to the GC T_FH_ phenotype (GL7^+^ T_FH_ cells)^14^, and sustain the development of an efficient GC response when compared to the young NP-specific T_FH_ cells. Thus, we propose that inadequate T-B cell interactions, as well as increased regulatory T cell number in aged mice contribute to age-related impairments in humoral responses to influenza infection (Supplemental Fig. 3).

## Materials and Methods

### Mice

Young (2–4 months) and aged (18–24 months) C57Bl/6 mice were bred and housed at the Trudeau Institute or University of Connecticut Health Center animal facilities. Alternatively, aged C57Bl/6 were obtained from the National Institute of Aging (NIA) aging colony bred and housed at Charles River Laboratories. Young mice were also obtained from Jackson Laboratories. All mice were kept in sterilized, individually ventilated, HEPA-filtered cages under specific pathogen-free conditions. The mice were anesthetized with isoflurane and infected intranasally with 600 PFU of the mouse-adapted influenza A H1N1 strain Puerto Rico/34/8 (PR8). The mice were sacrificed by CO_2_ asphyxiation. Age-matched non-infected mice were used as controls. The Trudeau Institute Institutional or University of Connecticut Health Center Animal Care and Use Committees approved all experimental procedures using animals and all procedures were carried out in accordance with their guidelines.

### Determination of antibody titers

Young and aged mice and were anesthetized with isoflurane and blood was harvested by cardiac puncture on days 0, 7, 14 and 21 post infection. The blood was allowed to coagulate for 2 hours at room temperature, centrifuged (×10,000 rpm) 10 min at room temperature, and the serum was collected and kept at −20 °C until analysis was performed. PR8-specific IgG was determined by ELISA using HRP-labeled anti-isotype antibodies (Southern Biotech Inc.). Antibody titers were determined by the last serum dilution with an optical density above background. Neutralizing antibody titers were determined using an *in vitro* neutralization assay. In brief, serum dilutions were incubated with 400 PFU of PR8 for one hour at 37 °C. The mixtures were added on Madin-Darby Canine Kidney cell monolayers and centrifuged (×3,000 rpm) 70 min at room temperature. The cells were next washed and incubated 20–24 hours at 33 °C in Zero-Serum Refeed Medium-PSGA (Diagnostic Hybrids) containing 4 μg/ml trypsin (Sigma). The virus was next detected using a biotin-labeled anti-influenza-A antibody (Millipore). The neutralizing antibody titers were determined as the last serum dilution with fewer viral plaques than the normal mouse serum control.

### Flow cytometry

To identify the NP-specific CD4^+^ T cells, single cell suspensions of splenocytes were first stained for one hour at room temperature with MHC class II NP Tetramer (PE/APC, Trudeau Institute Molecular Biology Core Facility). Further phenotyping was performed by staining with directly fluorochome conjugated antibodies (Supplemental Table 1). For intracellular transcription factor detection, cells were fixed and permeabilized using the Foxp3/Transcription Factor Staining Buffer Set (eBioscience) according to the manufacturer’s instructions. NP-specific GC B cells were detected by staining with PE labeled B cell tetramer (Trudeau Institute Molecular Biology Core^44^), for 30 min on ice. For intra-cellular cytokine staining, samples were enriched for CD4 cells by MACS^®^ using the CD4 enrichment kit II (Miltenyi Biotech) according to the manufacturer’s instruction. The cells were then resuspended at 5–10 × 10^6^ cells/ml in RPMI (Cellgro) containing 0.18 μM β-mercaptoethanol, 4 mM glutamine, antibiotic-antimycotic solution (Cellgro), 10 mM HEPES, 10% fetal bovine serum and 4 μl/6ml GolgiStop™ (BD). The cell suspensions were stimulated for 5 hours with 1 μg/ml phorbol 12-myristate 13-acetate and 1 μg/ml ionomycin at 37 °C. The cells were then washed, fixed, and permeabilized using the BD Cytofix/Cytoperm™ Fixation/Permeabilization Solution Kit (BD) according to the manufacturer’s instructions. Control samples containing the different “fluorescence minus one” antibody cocktails were used to determine the appropriate gating strategy for each cytokine. All flow cytometry samples were analyzed on a Canto II flow cytometer or on a LSR II flow cytometer using the FACSDiva software (BD). Data analyses were then performed using the FlowJo software (Tree Star).

### Immunofluoresence

Spleens were fixed overnight in medium containing 0.05 M phosphate buffer, 0.1 M l-lysine, pH 7.4, 2 mg/ml NaIO4, and 10 mg/ml paraformaldehyde and were dehydrated with 30% sucrose in phosphate buffer. Tissues were frozen in Tissue-Tek O.C.T. compound (Sakura Finetek) and sectioned using a CM1850 cryostat (Leica). 20-μm frozen sections were permeabilized with 1% Triton X-100 (Sigma) for ten minutes at room temperature, blocked for 30 minutes with Background Buster (Innovex Biosciences), stained over night at 4 °C in a humidified chamber with an antibody cocktail (Supplemental Table 2), then mounted with Immu-Mount (Thermo Sceintific). Immunofluorescence confocal microscopy was performed with the Zeiss 780 laser scanning microscope (Carl Zeiss; air objective 20× Plan-Apochromat with NA 0.5) using multichannel frame scans. The ZEN 2012 software (Carl Zeiss) was used for image acquisition. For half spleen images, the ZEN 2012 tile scan function was used to stitch individual 20x images together. Quantification of GC area, GC T_FH_ cells and image processing was performed using Imaris 8.1 software (BITPLANE).

### Adoptive transfer

The splenocytes were prepared from young non-infected CD45.1^+^ C57/Bl6 mice (Jackson Laboratories) and enriched for CD4^+^ cells by MACS^®^ enrichment kit II (Miltenyi Biotech) according to the manufacturer’s instruction. Purity of this population was confirmed by flow cytometry (>85%). The isolated young CD45.1^+^CD4^+^ T cells were tail vein injected into young or aged CD45.2^+^ C57/Bl6 hosts at 3.6 × 10^7^ cells/200 μl/ mouse. One day later the mice were infected with 600 PFU PR8 influenza and subsequently sacrificed 14 days later for flow cytometric analysis.

### Active TGF-β 1 ELISA

Prior to flow analysis, the spleens from young and aged mice that received adoptive transfer of young CD45.1^+^CD4^+^ T cells, where crushed through 100μM wire mesh with 400μL of PBS. The suspension was then centrifuged (×300 rpm) for 2 minutes and supernatant was collected and stored at −80 °C until analysis was performed. TGF-β1 was measured in the spleen supernatants using the LEGEND MAX^TM^ Free Active TGF-β1 ELISA kit (BioLegend). Samples were run in duplicate according to manufactures instructions. Spleen weights did not differ significantly in young and aged mice (data not shown).

### *In vitro* generation of regulatory T cells

A sterile single cell suspension was prepared from spleens and lymph nodes pooled from four young mice. Erythrocytes were lysed using ACK lysis buffer (Gibco by Life Technologies). CD4^+^ T cells were isolated by negative selection using Miltenyi’s CD4 cell isolation kit according to manufactures instructions. The CD4^+^ population was then stained with PE conjugated anti-CD25 (BD). After washing, the cells were then incubated with anti-PE microbeads (Miltenyi). CD25^+^ cells were removed by positive selection using the LS column (Miltenyi). Purity of the CD25^−^CD4^+^ T cells was confirmed by flow cytometry. The purified cells were suspended at 2 × 10^6^ cells/ml in *X-Vivo*15 serum free medium (Sartorius Stedim Biotech) and cultured in 24 well plates with Dynabeads^®^ Mouse T-Activator CD3/CD28 (Life Technologies) at a bead to cell ratio of 1:1. TGF-β1 (R&D Research Systems) was either added at 5000 pg/ml (recommended concentration for generation of regulatory T cells (Fantini *et al.*^35^) 100 pg/ml (concentration found in aged spleens), or 0 pg/ml (negative control). All conditions were plated in triplicate and cultured for 5 days at 37 °C 5%CO_2_.

### Statistical analyses

All experiments were performed at least twice, and each group contained 3–7 animals. Statistical significance was determined by Student’s t test, One-way or Two-way ANOVA, Repeated Measures ANOVA, or Pearson’s correlation as specified in the figure legends. Statistical analyses were performed with Prism 5 or Prism 6 software (GraphPad Software inc.) or SPSS Software (IBM). Differences were considered significant at p < 0.05.

## Additional Information

**How to cite this article**: Lefebvre, J. S. *et al.* Age-related impairment of humoral response to influenza is associated with changes in antigen specific T follicular helper cell responses. *Sci. Rep.* **6**, 25051; doi: 10.1038/srep25051 (2016).

## Supplementary Material

Supplementary Information
