# Supplementary material for: Age-related impairment of humoral response to influenza is associated with changes in antigen specific T follicular helper cell responses
Source: Sci Rep. 2016 Apr 25;6:25051. doi: 10.1038/srep25051 (PMC4842996; doi:10.1038/srep25051)
Supplement: Supplementary Information [file srep25051-s1.docx]

| **Antibody** | **Fluorochrome** | **Clone** | **Manufacturer** |
| --- | --- | --- | --- |
| CD4 | APC-eFluor®780  eFluor®450 | RM4-5 | eBioscience |
| GL7 | FITC | GL7 | BD |
| CXCR5 | PE-Dazzle^TM^594  BV421 | L138D7 | BioLegend |
| PD-1 | PE-Cy7 | RMP1-30 | BioLegend |
| CD44 | BV510 | IM7 | BioLegend |
| CD45 | AF700 | Dan11mag | BioLegend |
| CD45.1 | BV510  BV421 | A20 | BioLegend |
| ICOS | AF488 | C398.4A | BioLegend |
| Ly108 | PE | 13G3-19D | eBioscience |
| OX40 | PE | OX-86 | eBioscience |
| CD150 | PE | Q38-480 | BD |
| CD19 | BV650  PE-Cy7 | 6D5 | BioLegend |
| Bcl6 | PerCP-eFluor®710 | BCL-DWN | eBioscience |
| Foxp3 | eFluor®450 | FJK-16s | eBioscience |
| Peanut lectin agglutinin | FITC |  | Sigma |
| CD38 | Pacific Blue | 90 | BioLegend |
| IFN-γ | FITC | XMG1.2 | BD |
| IL-4 | PerCPCy5.5 | 11B11 | BD |
| IL-2 | APC-Cy7 | JES6-5H4 | BD |
| IL-10 | V450 | JES5-16E3 | BD |
| IL-21R Fc chimera  (IL-21 Primary) | Unconjugated |  | R&D Systems |
| Goat anti-human IgG Fcγ Frag Specific  (IL-21 Secondary) | PE |  | Jackson ImmunoResearch |
| Propidium iodide  (Live/Dead Indicator) |  |  | Sigma |
| Carboxylic Acid, Succinimidyl Ester  (Live/Dead Indicator) | AlexaFluor®350 |  | Life Technologies |
| CD16/CD32  (FC Block) | Unconjugated | 93 | eBioscience |

**Age-related impairment of humoral response to influenza is associated with changes in antigen specific T follicular helper cell responses**

*Julie S Lefebvre^1#^, *April R Masters^2^, Jacob W Hopkins^2^, and Laura Haynes^1#^

**SUPPLIMENTAL FIGURES**

**Supplemental Table 1. Antibodies used for phenotyping T cells, B cells and cytokine production by flow cytometry.**

**Supplemental Table 2. Antibodies used for confocal microscopy.**

| **Antibody** | **Fluorochrome** | **Clone** | **Manufacturer** |
| --- | --- | --- | --- |
| B220 | BV510 | RA36B2 | BioLegend |
| GL7 | AF488 | GL7 | BioLegend |
| CD4 | BV421 | GK1.5 | BD |
| Bcl6 | AF647 | K112-91 | BD |

**
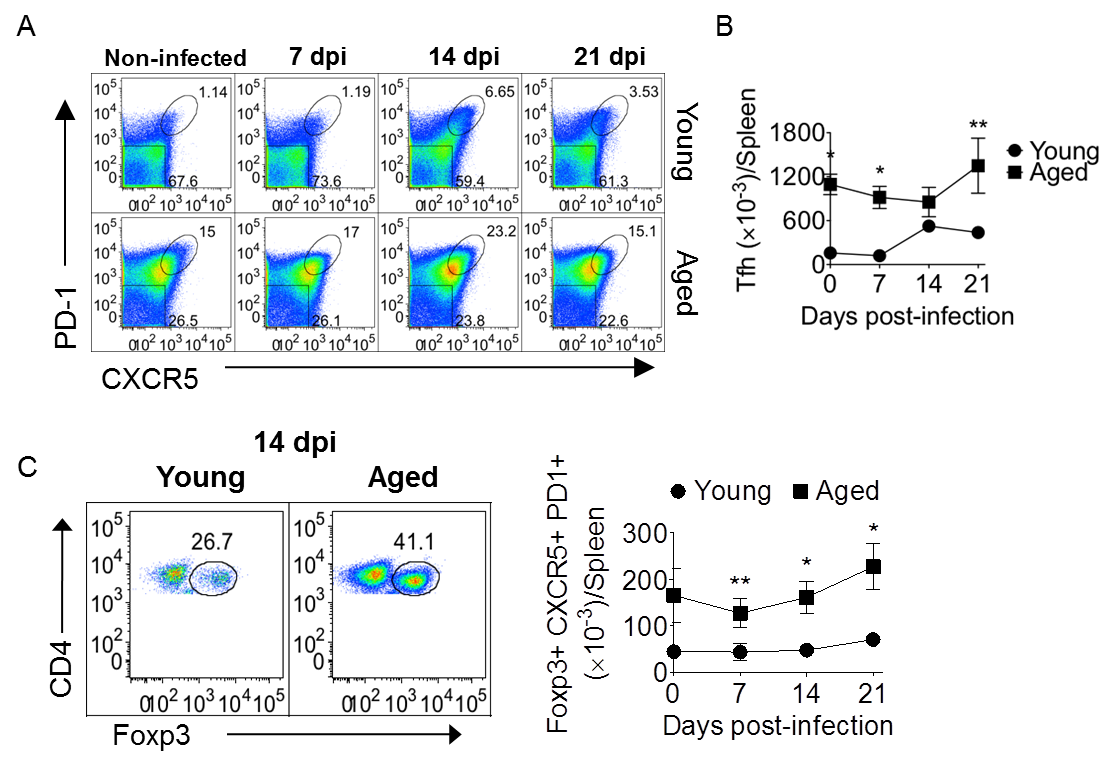
**

**Supplemental Figure 1. Increased number of T follicular helper cells and T follicular regulatory cells in aged mice compared to young mice.** Young and aged mice were infected with 600 pfu PR8 influenza virus. **(A)**Representative flow cytometry plots of T follicular helper cells from non-infected, day 7, day 14 and day 21 post infection were gated on alive, CD19-, CD4+ cells that expressed CXCR5 and PD-1. Total number of T follicular helper cells through the course of influenza infection (B). (C Left) The frequency of T follicular regulatory cells at 14 days post infection, gated as in figure A, with the addition of Foxp3. (C Right) Total number of T follicular regulatory cells in young and aged mice through the course of influenza infection. Data shown are one representative experiment with mean ± SEM of 5-8 mice/group from 3 independent experiments. Statistical significance was determined by two-way ANOVA followed by Bonferronni’s post tests.*, p<0.05,**, p<0.01.

**
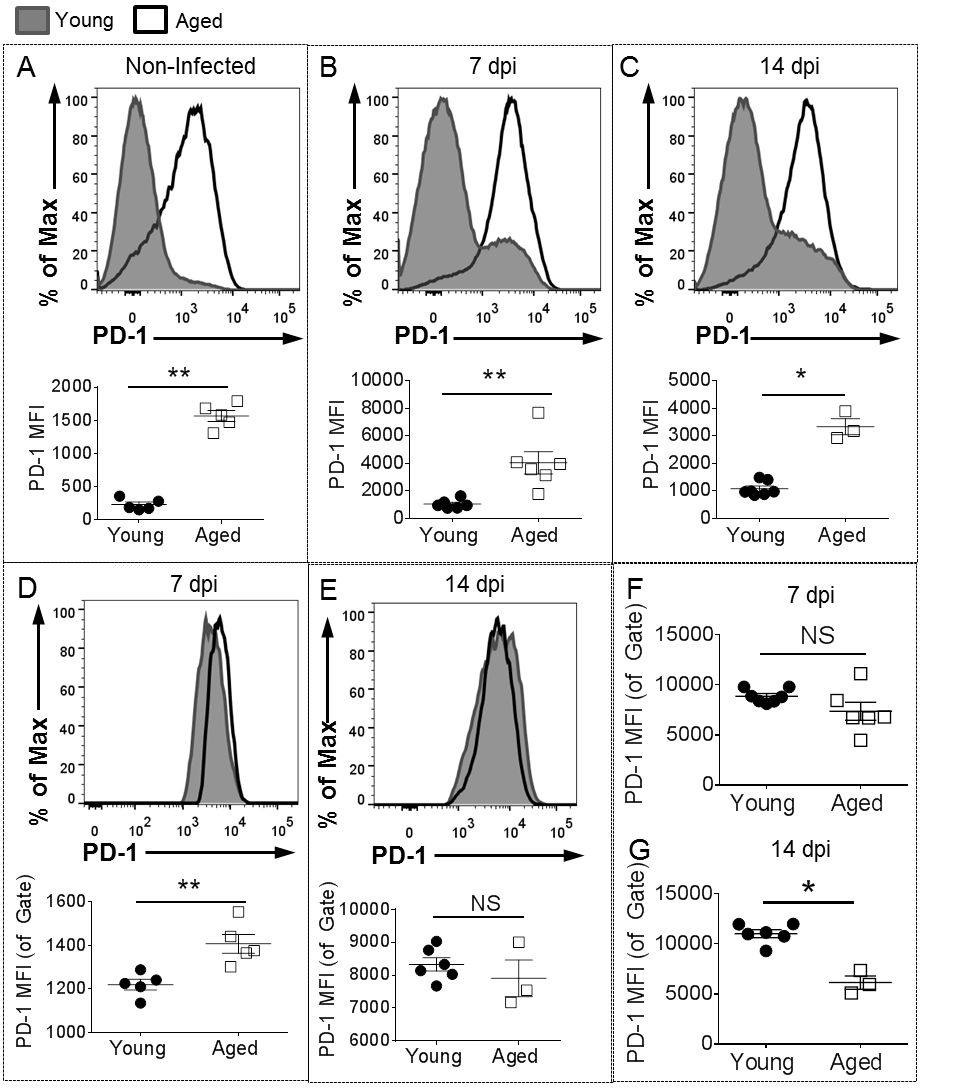
**

**Supplemental Figure 2. Differential expression of PD-1 on young and aged T follicular helper cells.** Young and aged mice were infected with 600 pfu PR8 influenza virus and the splenic response was determined at 7 and 14 days post infection. PD-1 MFI of total CD4+ T cells (gated on lymphocytes, single cells, alive, CD4+) of A) naïve mice, B) 7 days post infection and C) 14 days post infection. The PD-1 MFI of T follicular helper cells gated as described in Sup Fig 1. was determined in D) 7 days post infection and E)14 days post infection. The expression of PD-1 on NP Tetramer+ T follicular helper cells (as gated in Figure 2) was determined F) 7 days post infection and G)14 days post infection. Data shown are one representative experiment with mean ± SEM of 3-8 mice/group from 2 independent experiments. Statistical significance was determined by the Mann-Whitney Test.*, p<0.05,**, p<0.01.


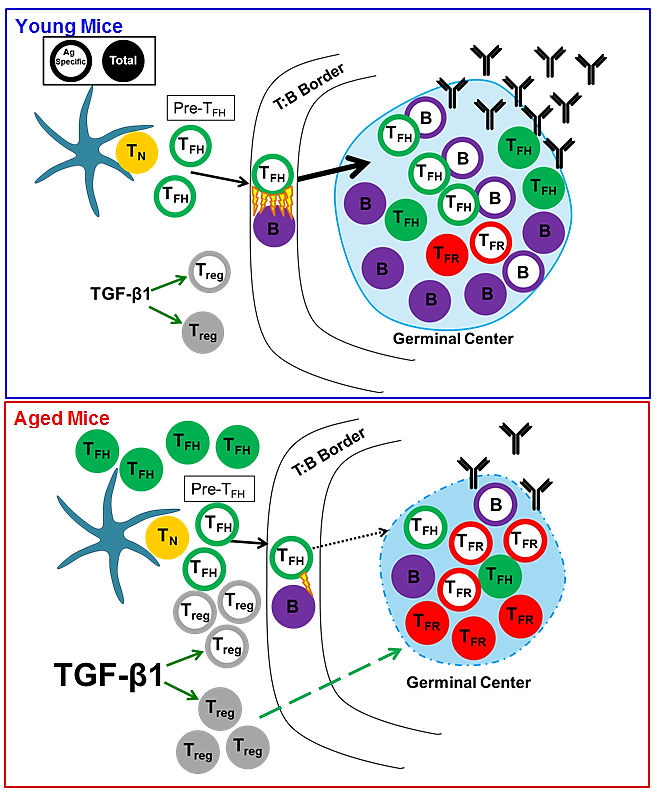


**Supplemental Figure 3. Graphical summary.**
